# A Systematic Review of Research Patterns and Trends in Early Childhood Education Teacher Well-Being from 1993 to 2023: A Trajectory Landscape

**DOI:** 10.3390/bs14121179

**Published:** 2024-12-10

**Authors:** Xinlin Li, Lutong Zhang, John Chi-Kin Lee, Junjun Chen

**Affiliations:** 1 School of Foreign Languages and International Business, Guangdong Mechanical and Electrical Polytechnic, No. 2 Chanchushi Rd. East, Baiyun District, Guangzhou 510515, China; cindyli@gdmec.edu.cn; 2 College of Applied Technology, Yunnan Minzu University, No. 134, Yi Er Yi Street, Wuhua District, Kunming 650000, China; 042036@ymu.edu.cn; 3 Department of Curriculum and Instruction, Academy for Educational Development and Innovation (AEDI), The Education University of Hong Kong, 10 Lo Ping Road, Tai Po, New Territories, Hong Kong, China; 4 Department of Education Policy and Leadership, The Education University of Hong Kong, 10 Lo Ping Road, Tai Po, New Territories, Hong Kong, China; jjchen@eduhk.hk

**Keywords:** teacher well-being, early childhood education, review of research, descriptive quantitative analysis

## Abstract

This current systematic review of research represents an attempt to unveil a descriptive picture of the evolving state of the body of knowledge about the well-being of early childhood education (ECE) teachers over 31 years from 1993 to 2023. In this review, 167 articles selected from SCOPUS are analysed using a descriptive quantitative approach. The analysis shows that quantitative research methods and empirical research are predominant in this field. Significant increases have been identified in qualitative and mixed-method research approaches in recent years, presaging a more balanced knowledge base in the near future. However, there has been little change in the scarcity of other research types. Meanwhile, the fragmented conceptualization of well-being, as well as its corresponding measurement, remains an outstanding issue. Overall, this review lays the groundwork for an understanding of the current trajectory of well-being research, especially with respect to ECE teachers, from a developmental perspective.

## 1. Introduction

Many early childhood education (ECE) teachers enter the profession with high enthusiasm and expectation, with a sense of being called to take good care of the future generation. However, the real work situation is rather challenging even with such strong commitment [1]. Research shows that many ECE teachers are suffering from higher levels of negative experiences than people in other professions [2,3]. In comparison to other educational levels, ECE teachers are also more physically and emotionally drained [3,4]. As a result, turnover rates of ECE teachers are some of the highest in the education industry. In the USA, around 30% of ECE teachers leave their jobs annually, which is higher than the rates of other levels [5,6]. Likewise, turnover rates in other countries like China have been hovering around 30%, causing costly consequences for all stakeholders [7]. Interpretation of the term well-being may vary significantly. Nonetheless, it consistently refers to a more positive state that people strive to achieve, encompassing aspects such as physical and mental health, professional wellness, and more [8]. For a long time, it has usually been assumed that teachers are able to manage their own well-being, including those in the ECE profession, which has therefore resulted in a lack of support for ECE teachers [8]. Also, it appears that many teachers are encouraged to prioritise instruction quality and children’s performance over their own well-being, which has adversely impaired teachers’ effectiveness and sustainability and resulted in underestimation of the cruciality of teacher well-being [9].

Since 2020, the COVID-19 pandemic has imposed further pressure onto this already strained profession. Increased symptoms of poor mental health have become evident among all teachers, including those who have been entrusted with the most vulnerable young children, namely, the ECE workforce [10,11]. Ironically, it is during such a crisis that the role of a sense of well-being can be better appreciated, studied, and promoted. In addition, an insufficient number of potential student candidates, coupled with higher difficulty of recruiting students, has become more common and created new risks for teachers. These challenges have brought the threat of job insecurity, as well as higher job demands, which may therefore exacerbate ECE teachers’ stress and impact their work engagement [12,13].

The significance of promoting teacher well-being is multi-fold, which highlights the necessity of investigating this topic. First, it has been well acknowledged that teacher well-being is associated with teachers’ intention to stay or leave their positions [14]. Even before departure, teachers with intents to leave have been proven to be less engaged in their work, leading to poorer instruction quality. Beyond the individual level, teacher well-being is key to the retention of a quality ECE workforce, namely, the sound and sustainable development of children and schools [15,16]. For instance, in Silver and Zinsser’s [17] study, teachers with better well-being were less likely to give expulsion orders to children. According to Kwon et al. [18], children’s problematic behaviour might be the result of reacting to and modelling teachers’ depression symptoms. Meanwhile, at the school level, an unstable and dissatisfied workforce might cost schools continued investment to remedy the “disrupted continuity of instructional program” and to “mentor and assist new teachers” [19]. Notably, during this challenging period—particularly in regions (e.g., in the USA and China) facing declining student enrolment and the threat of kindergarten shutdowns—there is a more urgent need to enhance the quality of ECE teachers, optimise educational resources, and strengthen competitiveness [12]. Thus, investing in the well-being of ECE teachers can be a crucial strategy for kindergartens to navigate this crisis and to position these teachers for long-term sustainability.

With the growing recognition of ECE teacher well-being, it is encouraging to see that publication in the field has been amplified in recent years. As the intellectual knowledge of a field accrues, research reviews play a critical role by synthesising what is currently known about the topic and informing future research directions [20]. Therefore, this systematic review aims to comprehensively examine the existing knowledge base on ECE teacher well-being from a broader and developmental perspective. By doing so, it seeks to identify the trajectory of research patterns and trends in the field partially based on some previous work from other scholars [9,21] and our team members [22,23,24] to generate reflections on future research directions and implications for practice.

## 2. A Historical Overview of Research on ECE Teacher Well-Being

One of the early references to the term “well-being” appeared in the World Health Organization’s [25] constitution, which defines health as “a state of complete physical, mental and social wellbeing and not merely the absence of disease or infirmity”. Nowadays, the term well-being is ubiquitous but has proven to be hard to be defined. Recognising the semantic breadth of well-being, many researchers even choose not to establish a firm definition in their studies. For the past few decades, literature examining well-being has been dominated by studies of several related and more specific constructs, such as stress, burnout, and job (dis)satisfaction, mostly seeking answers to what makes people depressed and how people can overcome negative experiences [9]. Around the turn of the twenty-first century, Seligman [26] coined the term “positive psychology” to highlight the power of bolstering well-being within teachers, encouraging more researchers to pay attention to the construct “well-being” itself instead of any other prevalent element. At present, despite the diverse interpretations, well-being is commonly viewed as “something we all aim for, underpinned by positive notions”, and, more importantly, as something that varies across different persons [8]. Practically, the OECD [27] has defined well-being as “multidimensional, covering aspects of life ranging from civic engagement to housing, from household income to work-life balance, and from skills to health status”. In the context of educators, well-being primarily focuses on how teachers respond to various conditions within their work and profession [28].

It is interesting to note that a substantial amount of literature related to “well-being” actually focuses on student well-being, investigating how schools and teachers can fulfil students’ needs [29,30]. After all, the concern for students’ growth always lies at the heart of education. The situation has been gradually transformed by an intensified interest in connecting students’ well-being and teachers’ well-being and in the high turnover rates and intentions among teachers around the globe [31]. It has been suggested that with lower well-being states, teachers are less likely to place sufficient attention on instruction and are more prone to resign from their positions [1,14]. As per a study in Finland, around half of teachers had plans to resign, and the percentage was remarkably persistent over five consecutive years [32].

Among all educational levels, turnover rates in ECE remain at a high level due to some common challenges such as work overload and relationships with parents. In addition, factors like physical discomforts, insufficient governmental support, payment, and social recognition characterise the profession [3,33,34]. Comparatively, the development of ECE has been slightly neglected. Currently, many ECE educators are still struggling with low pay and low job status [35]. One potential reason for undervaluing this profession is the deep-rooted impression of the babysitting role of ECE teachers [36]. However, along with a growing importance attached to ECE for children’s growth, ECE teachers have been urged to step far beyond being simply “nurses” or “carers”. Although ECE teachers commonly report that they enjoy working with children, they are exhausted by multiple stressors, such as the escalating requirements from parents and societies, as well as increasing legislative demands from accreditation systems. Unfortunately, many of them receive little support from their education background and working conditions [37]. In other words, many ECE teachers suffer from the expanding gap between resources and constantly growing job demands [3], thus contributing to the reported high pressure that they experience and the escalation of publications focusing on maintaining and protecting their well-being.

## 3. Previous Reviews on ECE Teacher Well-Being

Prior to the current review, there have been several foundational review papers summarising what has been explored and detailing what can be expected. For instance, in the very first review aiming at ECE teacher well-being, a total of 30 articles from various locations were identified from ERIC, Google Scholar, and other prominent journals and websites using the following keywords: early childhood/preschool teacher well-being; job satisfaction; stress; burnout; and teacher quality [9]. This review mainly focuses on the contents and methodologies of the selected literature. For example, the authors identified several frequently observed themes, such as compensation, educational qualifications, job satisfaction, and job stress, as well as some emerging topics, such as self-efficacy and life satisfaction. They also noted the gap regarding methodologies in the field, such as the imbalanced geographical distribution, limited measurements, and participants. This review represents the first vital step to describe the research landscape on ECE teacher well-being in a global context and thus can be seen as a significant milestone in this field. However, this review also has limitations. Due to the relatively small number of selected and fragmented themes, this review was unable to provide a comprehensive profile of the subject. Also, it should be noted that the authors excluded caregivers (birth-3) from the sample of preschool teachers due to their different career requirements as observed in the USA where most relevant research has occurred.

The next important review by Cumming [21] analysed another 30 papers with basically the same pattern as the 2014 review [9] (i.e., similar criteria and review objectives). In addition to the previous keywords used, the author provided an alternative keyword, “educator”, in place of “teacher” as she sensed the nuance between education systems in the USA and other regions (e.g., Australia). In the same vein, Cumming also included the previously excluded group, caregivers, to achieve a more comprehensive examination. This review effectively complements the previous review by illustrating changes regarding themes and methodologies over time. Slightly different from findings of the last review, researchers appear to have focused less on the impacts of compensation. Instead, the work environment has become more mainstream. The author also noted some improvements in methodological issues, such as the diversification of locations for data collection and measurement tools, while the conceptualisation of well-being has remained rather elusive.

As the literature on ECE teacher well-being has begun to take shape, more reviews have continued to emerge to showcase the field’s progress, although with different emphases. For example, the study by Fenech et al. [38] centred on the theoretical framing of ECE teacher well-being with a focus on data from Australia. Berger et al. [39] concentrated on interventions to facilitate teachers’ well-being. In contrast, a review by Wilson et al. [40] exclusively examined research on head start teachers rather than the ECE teaching population. The current review, therefore, represents a timely effort to conduct a more comprehensive examination of research attempts concerning ECE teacher well-being. This includes an analysis of the developmental patterns of publication years, research types, data collection locations, research foci, and research methods. Also, informed by previous reviews, the researchers purposefully included all teachers of children younger than school age from various potential areas in search of a clearer and impartial portrayal of the knowledge base in the field. To achieve the goal, three research questions are proposed below to guide the following analysis and discussion:

1. How has the body of research on ECE teacher well-being evolved from 1993 to 2023, particularly regarding annual publication numbers, data collection locations, and research topics?
2. How has the distribution of research types in the field, including empirical studies, reviews, and conceptual and commentary papers, changed between 1993 and 2023?
3. How has the use of research methods in the ECE teacher well-being literature changed from 1993 to 2023?

## 4. Method

Under the guidance of the Preferred Reporting Items for Systematic Review and Meta-Analysis (PRISMA) [41], the authors completed the procedures of setting up the criteria, searching for and identifying relevant articles, screening those articles, extracting data, and finally, analysing the data. To further ensure the robustness of the process, the researchers completed registration of this review on Open Science Framework.

### 4.1. Inclusion and Exclusion Criteria

There were five inclusion criteria for identifying potentially qualified documents. First, the document was required to be related to “teacher well-being” or relevant indicators. Second, it had to include ECE teachers as participants. Third, the document must have undergone a peer review process. Fourth, it needed to be presented in English. Fifth, the document was required to fall within one of the following research types: empirical studies, reviews, and conceptual or commentary articles.

In addition, there were three exclusion criteria to ensure eligibility. First, if ECE teacher well-being was not part of the stated purpose, the document was excluded. Second, if ECE teachers were not explicitly stated to have served as participants, the document was excluded. Third, if the main text was not in English, the document was excluded.

### 4.2. Literature Search and Study Identification

The current review focused on relevant journal articles on ECE teacher well-being published between 1993 and 2023. This period of 31 years was selected for empirical, historical, and pragmatic reasons. First, a previous review identified a time range beginning around 1990 for this topic [9], establishing the preliminary reference for the time range of this review as well. Further, a historical perspective of this field indicates that although studies about related constructs of well-being can be traced to decades ago, the wave of studies dedicated to “well-being” itself was initiated around the beginning of the twenty-first century. Third, the earliest publication retrieved from the initial literature search for this review was from 1993. Thus, the time range was set from 1993 to 2023, covering a period of 31 years.

The search process commenced with a systematic inquiry in SCOPUS in June 2024, with the keyword string “educator OR teach* AND well-being OR wellbeing”. In cases of any missing qualified, significant document or a risk of publication bias, Google Scholar and a “snowball strategy” were implemented. First, the authors searched the same keywords in the database of Google Scholar to cover grey literature [42]. Second, the authors searched through the reference lists of previous important review articles [43]. At the end of this stage, the authors retrieved a total of 14,447 articles for further screening.

### 4.3. Screening and Extraction

**Figure 1:**
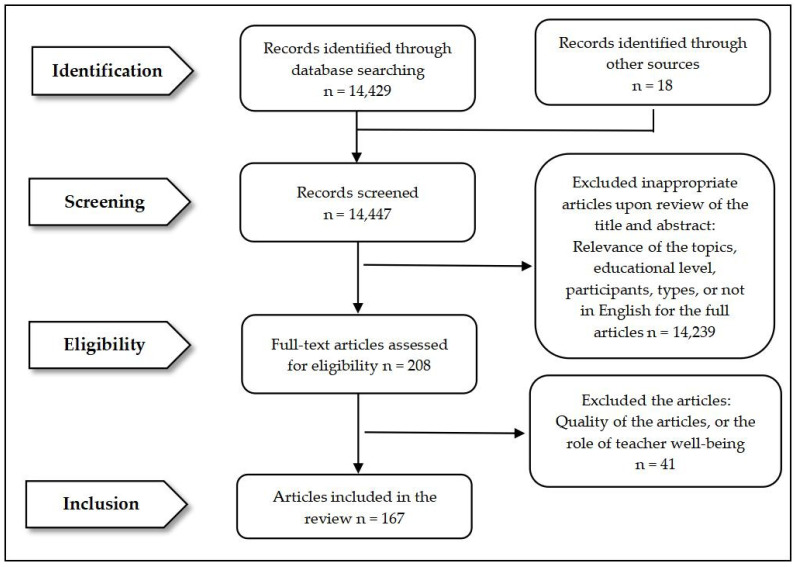
Search and screening process.

The retrieved articles underwent a four-step screening process to determine their compliance with the inclusion and exclusion criteria [41,42]. First, a total of 14,447 articles were initially identified through database searches. Second, one author reviewed all abstracts to select articles that met the inclusion criteria (e.g., topic relevance, educational level, and participants). In more detail, papers that actually focused on how teachers could enhance students’ well-being were excluded at this step [44]. If there were uncertainties about whether to include an article, another author evaluated the full text to arrive at a mutual decision. After this step, the authors selected 208 articles for further consideration. Third, the same author retrieved the full texts of the 208 articles and read through these materials to evaluate their quality and the centrality of teacher well-being. Similarly, any uncertainties were resolved through joint efforts and discussions between two or more authors to achieve consensus. A third researcher joined the discussion when the two researchers failed to reach a consensus. Forty-one articles were then excluded because of poor quality, unsuitable participants, or teacher well-being having a marginal role in the study. Finally, 10 (10%) articles were randomly selected and checked a second time to verify the final selection. After all these steps, 167 articles were ultimately included (see Figure 1 for the process).

### 4.4. Analysis

In this review, a descriptive quantitative analysis approach was adopted to outline the changes and developmental trends over the specified time range [45]. The authors coded the qualified papers with the guidance of the data extraction instrument of Hallinger and Chen [20]. In addition to support from previous literature reviews, this instrument was initially evaluated on five papers, and the coding was refined accordingly. Subsequently, this adapted instrument guided the researchers to focus on several major aspects, including publication number, data collection location, research foci, research type, and research method. Throughout the process, inter-rater reliability of 80% was achieved across coding members.

## 5. Results

After the steps described above, 167 articles were finally included, including 159 (95.2%) empirical research articles, 6 (3.6%) review articles, and 2 (1.2%) commentary articles. As the selected time range covered 31 years, all data were clustered and calculated in 5-year segments to facilitate understanding and underscore the trends. It should be emphasised that not all papers were analysed for data collection locations (e.g., no location information) and research methods (e.g., commentary papers).

### 5.1. Publication Numbers

**Figure 2:**
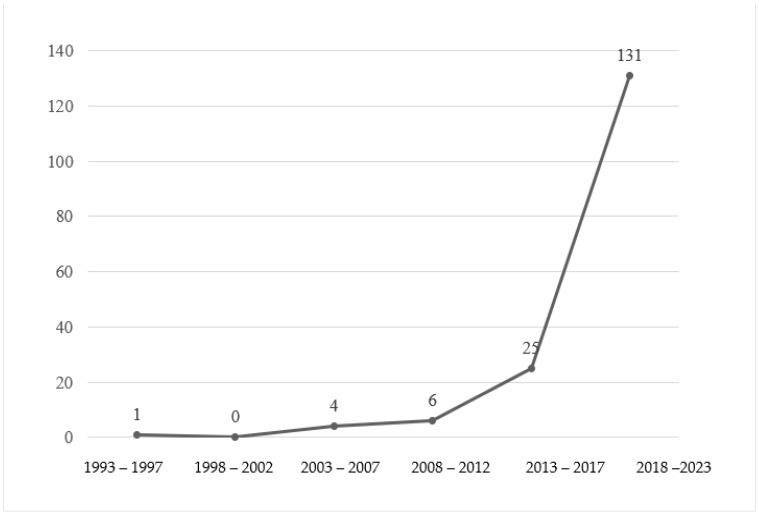
Publication numbers in five-year segments from 1993 to 2023 (N = 167).

Publication numbers from 1993 to 2023 showed an increasing trend (Figure 2). During the first four intervals (1993–2012), the literature on ECE teacher well-being grew slowly, averaging fewer than one article for every five years. The volume began to increase at a faster and more visible rate during the fifth interval (2013–2017), where the annual publication rate first approached two digits (8 each in 2016 and 2017). The latest interval (2018–2023) showed the fastest increase with 131 published papers, accounting for 78.4% of the total volume. Basically, the literature on ECE teacher well-being developed at a rather slow and steady rate during the first 20 years and then increased significantly in the last 11 years.

### 5.2. Data Collection Locations

After excluding papers that did not involve or specify any data collection details, the researchers analysed the data collection locations of the remaining 158 articles (94.6%). A total of 26 countries and regions were identified, and it was easy to spot the uneven development among these locations. Table 1 illustrates the trajectories of 10 countries and regions with the most publications. Research conducted in the top two regions, the USA (65, 41.1%) and Mainland China (18, 11.4%), accounted for over half of the selected papers. Three countries and regions, Hong Kong (16, 10.1%), Australia (13, 8.2%), and Finland (9, 5.7%), together accounted for around a quarter of the research volume. The remaining research came from the other 21 regions around the globe. In addition to the imbalanced distribution, it appeared that only eight regions demonstrated positive and growing trends (e.g., USA, Hong Kong, and Australia), while trends in other regions remained flat or unstable. Meanwhile, 12 countries and regions produced relevant knowledge only in the last 6 years (e.g., Mainland China, South Korea, and Ghana).

### 5.3. Research Foci

According to the extraction results, the selected papers were categorised in two levels (Table 2). At the upper level, there were three research foci: the nature of ECE teacher well-being (29, 18.2%), the antecedents of ECE teacher well-being (128, 80.5%), and the effect of ECE teacher well-being (48, 30.2%). Any single paper could cover more than one research focus. To be more intensive, the upper-level topics were further categorised into various sub-themes to exhibit researchers’ interest. For the cluster of papers studying the nature of well-being, four themes were derived: theory (5, 3.1%) (e.g., Self-Determination Theory) [46], definition (3, 1.9%) (e.g., work-related well-being) [47], content 19, 11.9%) (e.g., depression, stress, and motivation) [48], and measures (3, 1.9%) (e.g., Four-item Spiritual Well-being Index) [49]. Under the foci of teacher well-being antecedents, the three themes were contextual factors (24, 15.1%) (e.g., educational policy) [50], school-level factors (94, 59.1%) (e.g., organizational support) [16], and personal factors (78, 49.1%) (e.g., personal beliefs) [51]. Five second-level themes were recognised under the foci of effect: teachers’ personal outcomes (24, 15.1%) (e.g., teacher turnover) [52], teaching practices (13, 8.2%) (e.g., teachers’ expulsion orders given to children during lessons) [17], student learning processes (18, 11.3%) (e.g., children’s school readiness) [53], students’ academic outcomes (5, 3.1%) (e.g., children literacy) [54], and school-level outcomes (3, 1.9%) (e.g., school turnover rate) [15].

**Figure 3:**
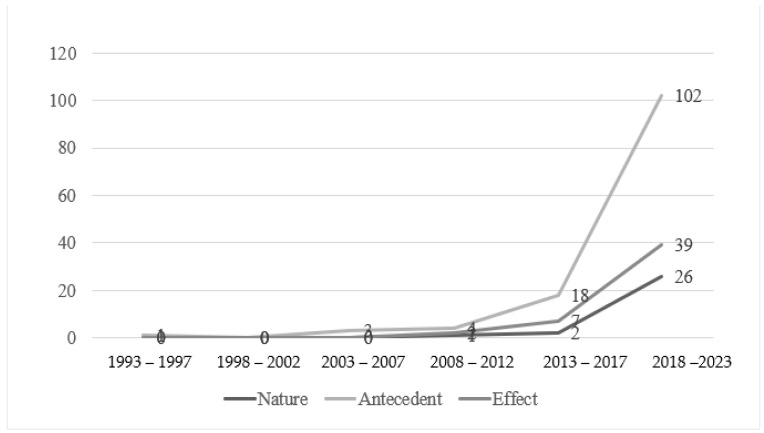
Research foci in five-year segments from 1993 to 2023 (N = 159).

Notably, compared with antecedent- and effect-related research, research exploring the nature of ECE teacher well-being stayed at a low level until the sixth interval (2018–2023) (Figure 3). Among the four sub-themes, a sharp discrepancy existed. Research studying the content of well-being accounted for half of the literature on this topic (18 out of 29, 62.1%), outnumbering the sum of the other themes.

The positive trend for antecedent-related papers started earlier, emerging in the third interval (2003–2007). Publications related to the four themes all increased, with the majority focusing on school-level factors (94 papers, 59.1%), which accounted for the largest share and showed the most rapid growth in this area.

Research about the consequences of ECE teacher well-being also emerged earlier, with its accumulation beginning in the fourth interval (2008–2012). Among the diverse effects, findings about teachers’ personal outcomes (e.g., turnover intentions) were prioritised (24, 15.1%), followed by students’ learning processes (18, 11.3%) and teaching processes (13, 8.2%). Research focusing on students’ academic outcomes (5, 3.1%) and school-level outcomes (3, 1.9%) received the least attention and continued to fluctuate at a lower level.

### 5.4. Research Types

**Figure 4:**
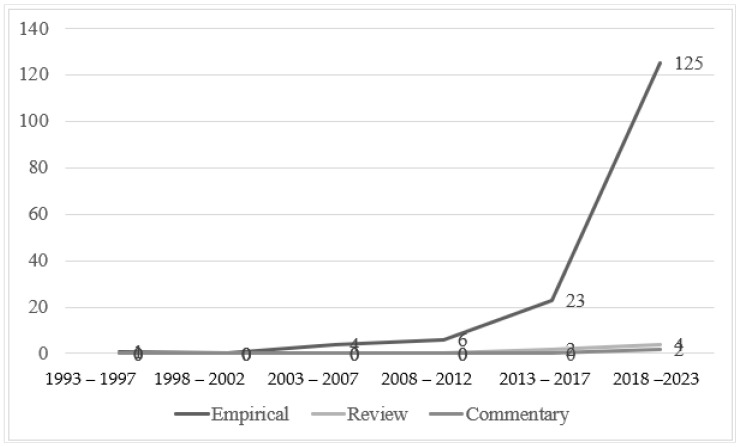
Research types in five-year segments from 1993 to 2023 (N = 167).

All research papers were categorised into three types: empirical articles, review articles, and commentary papers. Over the past 31 years, empirical articles dominated this topic (159, 95.2%), while review articles (6, 3.6%) and commentary papers (2, 1.2%) occupied very minor segments. Apart from this proportion, a positive trend can also be seen in the development of empirical articles beginning in the fourth interval (2008–2012) (Figure 4). Subsequently, empirical articles more than tripled across three consecutive intervals. In contrast to that of empirical articles, there was no output at all for the two other types of research until the last two intervals (2013–2023).

### 5.5. Research Methods

The research methods used in the selected papers were analysed in terms of (a) qualitative/quantitative/mixed methods, (b) quantitative statistical analysis level, and (c) qualitative data collection methods.

**Figure 5:**
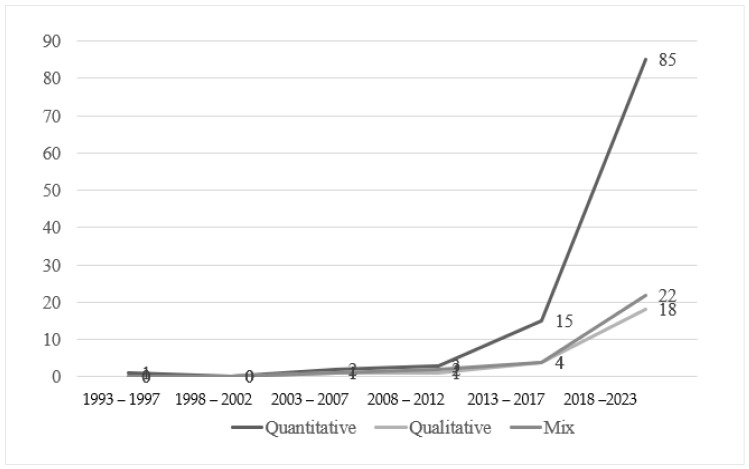
Research methods in five-year segments from 1993 to 2023 (n = 159).

First, the documents were coded according to the research methods used. From 1993 to 2023, researchers showed a preference for quantitative methods (106, 66.7%), followed by mixed-method approaches (29, 18.2%). Qualitative methods were the least favoured (24, 15.1%). Despite the disparities in amounts, positive tendencies were identified for all three research methods (Figure 5). Specifically, this upward tendency emerged the earliest among quantitative papers beginning in the fourth interval (2008–2012), with an accelerating speed thereafter. By contrast, the numbers of research articles using these two approaches also developed at a much slower pace. What is equally intriguing is the strong position held by non-experimental research. Specifically, among the empirical articles, only 28 articles (18.1%) used experimental designs, while 16 (10.3%) used integrated interventions.

Second, regarding the quantitative and mixed-method articles, the researchers further classified those papers according to the highest statistical analysis levels that the authors had reached. The sorting was based on a modified scheme of Hallinger [55] and Chen and Cheng [23] as follows:

Level 1: Descriptive analysis, focusing on central tendencies and/or the variability of scores.
Level 2: Single-causal factor analysis–correlational, focusing on relationships or associations between two variables.
Level 3: Single-causal factor analysis–correlational with controls, focusing on relationships between two variables while controlling for the influence of one or more other variables.
Level 4: Multiple-factor analysis, focusing on differing effects of multiple sources of influence on a specific variable.
Level 5: Advanced modelling analysis, focusing on relationships among several independent and dependent variables, allowing examination of moderating and/or mediating effects.

**Figure 6:**
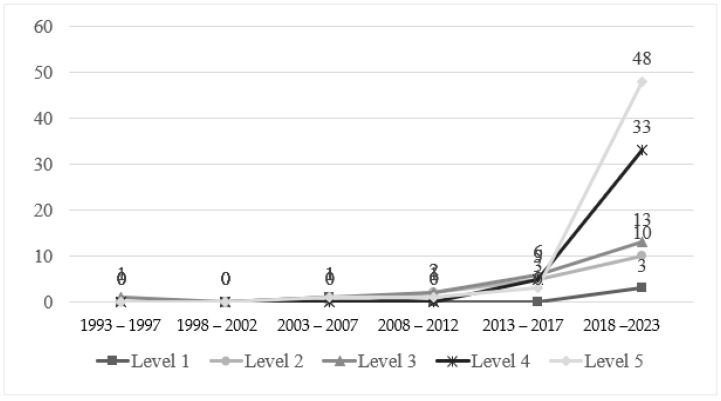
Statistical levels in five-year segments from 1993 to 2023 (N = 135).

Figure 6 shows that Level 5 statistics (53, 39.3%) were utilised most frequently, especially in the latest interval (2018–2023), followed by Level 4 (38, 28.1%), Level 3 (23, 17%), Level 2 (17, 12.6%), and Level 1 (4, 3%). Level 5 and Level 4 were the most rapidly increasing technique levels among the five. They increased by more than 10 times and 5 times, respectively, in the last interval (2018–2023). It is also noteworthy that 105 (77.8% out of 135 quantitative and mixed-method papers) adopted cross-sectional research designs, while only 32 articles (23.7%) used longitudinal designs.

Finally, 53 qualitative articles and mixed-method articles were analysed based on their qualitative data collection methods (Table 3). Interviews (25, 47.2% out of 53) and classroom observations (12, 22.6%) were the two most popular techniques. Techniques ranked in the second tier were focus groups (9, 17%), qualitative surveys (4, 7.5%), and case studies (3, 5.7%). Journal writing, life experience, and reflective reports were each used once only (1, 1.9%). From a developmental perspective, qualitative methods were seldom adopted in the first 15 years (1993–2007). The fourth period (2008–2012) was when more researchers began to adopt qualitative approaches to study ECE teacher well-being at deeper levels. Until that point, positive trends seemed to be noticeable and stable for only three techniques: interviews, classroom observations, and focus groups.

## 6. Discussion

Based on the findings above, this section will present a discussion of the major findings and their implications for future research foci and research designs.

First, there has been a significant increase in publication volume in the past 11 years. This trend, in some ways, echoes the changing understanding and conceptualisation of this construct. Historically, more emphasis has been placed on elements like stress and burnout, with rumination on how to free teachers from illnesses and disorders [56]. Later, as recognition of ECE teacher well-being increased, proposals were made to improve the working conditions in ECE to ensure teachers’ better well-being status [57]. The focus also began to shift from saving teachers from negative experiences to bolstering well-being within teachers, drawing more attention to the full concept of well-being [58]. The COVID-19 pandemic further sparked passion for this construct to better support the strained ECE teachers amidst the chaos. In fact, publications in the last period (2018–2023) contributed 78.4% of the total volume. However, the authors also note a dip in 2023, which cautions researchers to more carefully observe and consider the future development of the field. More time is needed to determine whether this is simply a numerical fluctuation as in previous years or a sign of the fading impact of major social crises like the pandemic.

Second, as in some previous studies, the literature in this field has been led by more developed western societies, signalling a gap between these regions and others in academic resources and focus received [21]. Their leading economic resources may be one driving force, and the abundant within-nation variations facilitated the preliminary exploration. While such endeavours contributed to the current knowledge base, the over-focus on certain countries constrained the diversity and the emergence of a worldwide intellectual structure [20,59]. Nevertheless, it is still encouraging to witness a more internationally diverse spread in the current review than those in previous ones, portending richer data resources for a more balanced intellectual framework. To address the gap, cooperation between different countries and regions should be encouraged as a means to promote circulation of ideas globally and bridge the disparities between regions [21]. At the same time, more comparisons may be promoted to advance the understanding of context-sensitive well-being. Researchers should treat well-being in these socially defined contexts with more discretion [60].

Third, the current review adopted a comprehensive and developmental lens and observed both convergent and divergent features of the knowledge base. In a way, the studies have centred around three major directions, namely, nature, antecedents, and effects of ECE teacher well-being, to build up a systematic understanding of the field. From another angle, the field has also diverged with a number of sub-themes to enrich the knowledge base, but in an imbalanced manner. For instance, the low share of research into the nature of well-being (18.2%) indicated a gap with insufficient attention, echoing the need to probe into the complexity of well-being to mitigate its fragmented conceptualisation [9,21,38]. To mitigate the imbalance, more discussions should focus on well-being’s other aspects, such as consequences and teachers’ unique interpretations of well-being at different career stages. Such endeavours could lead the field to have a more balanced topic coverage and hence a more mature and solid knowledge base [20].

Fourth, the disparity between empirical research and other research types was not unexpected given the small volume and the initial stage of the scholarship. It is believed that substantial accumulation of high-quality empirical research is essential for the construction of a sound knowledge base [20]. Nevertheless, the lack of conceptual and commentary papers indicates a need for greater effort in the future. In other words, there should be a stronger emphasis on theoretical discussions to construct and clarify the concept of ECE teacher well-being.

Fifth, quantitative studies were the most prevalent across all time periods. This is rather surprising as qualitative research methods normally take the lead in the preliminary stage of a field to establish a firm theoretical base for subsequent development. Apart from this type, most research adopted a non-experimental approach (111, 71.6%), which may adversely constrain the advancement of effective strategies and well-being-related causal relationships and thus limit the influences in the real world [61]. Thus, several pieces of advice could be given here to rectify the gap. Firstly, qualitative and mixed-method research designs should receive more attention. Recognising the parallel trends of research designs and research foci, the promotion of less commonly used approach could lead to deeper exploration and clearer understanding of the complex concept. Additionally, the use of mixed method design may foster innovation and enhance the credibility of future research, laying a foundation for development of other aspects, such as measurement design [62]. Secondly, a wider variety of empirical research designs, including experimental designs, is encouraged. This approach can yield more significant impacts in the real world with effective strategies to support teacher well-being [61].

Sixth, the quantitative trends in terms of the statistical analysis level point to a growing capacity for conducting quantitative research. Interestingly, most articles using advanced modelling adopted cross-sectional design, reflecting some sort of binding between certain research foci and research design. Also worthy of note were the fact that while over 100 different quantitative techniques were identified, only 14 of them were designed exclusively for well-being. Another concern dependent upon the measurement was the tension between subjectivity and objectivity in terms of substance and assessment [63]. Concerns have long been raised regarding the validity and reliability of the extensive use of self-assessment tools. The intangibility and changeability of well-being further compounds the situation, as some scholars believe that self-reports on well-being could be influenced, at least partially, by transitory and some external contextual factors [64,65]. On the other hand, subjective measures are indispensable to understand attitudinal matters or judgement as a whole [63]. Due to the manifold nature of well-being, it is hardly surprising to see a lot of measurements ranging from the most subjective measures (subjective substance and assessment, e.g., self-evaluation of satisfaction) [66] to the most objective ones (objective substance and assessment, e.g., counting of absent days) [67]. Such variety, to some extent, obstructs the circulation and development of teacher well-being. Two major recommendations are therefore made to address these concerns. Firstly, in view of the lack of specific scales, more effort could be made to design or validate well-being-focused measurement tools. Particular attention could be given to a meaningful combination of subjectivity and objectivity (e.g., first-person lens and objective indicators) when developing a new tool. Secondly, the changeable nature of well-being also inspires more complex research design, such as longitudinal design. In light of the predominating cross-sectional studies, research designs documenting changes over-time are needed to capture the dynamic nature of teacher well-being.

Seventh, the distribution of qualitative data collection was highly skewed in the direction of interview and classroom observation. Innovative techniques are lacking, except for some attempts in quantitative research, such as body mass index [68], salivary cortisol and alpha-amylase measurements [69]. Several recommendations are proposed for a more balanced development. Firstly, more innovative qualitative approaches are expected, not only to address the quantity gap, but also to facilitate a deeper exploration into the nature of teacher well-being at ECE level. Secondly, it is delighted to witness that a retrospective technique (e.g., interviews) and a more instant technique (e.g., classroom observations) were both valued by researchers. Nonetheless, the combination of these approaches has been less frequently employed, which could potentially allow research to transcend original limitations and examine the topic from multiple perspectives.

## 7. Limitations

Irrespective of the potential contribution, there are certain limitations of the current review. Firstly, SCOPUS was the only database to search for peer-reviewed papers, hence papers exclusive to other databases or other sources might be left out. However, the researchers are confident that most papers on teacher well-being have been included due to the high coverage of quality journals of social science in SCOPUS. In addition, with two recommended strategies, namely Google Scholar and Snowball strategy, omitted papers would make little difference to the analysis results. Secondly, the authors managed to include all papers involving the ECE workforce manually and did not specify age range for the children. To some extent, this might cause some confusion for comparison when targeting more specific educational periods especially between different educational systems. Future researchers may consider more refined criteria for particular research purposes. Thirdly, this review was designed to describe the developing trends and patterns rather than to more detailed analyses of individual articles, such as specific antecedents, consequences, or interventions used. Therefore, no explicit content discussion is presented in this attempt. More in-depth analysis concentrating on topic themes is expected in future efforts. Also, it should be encouraged to examine ECE teacher well-being from a variety of sociological lenses such as the feminist perspective to situate and deconstruct well-being in different social roles to generate deeper insight. Fourthly, the researchers made the decision with full awareness to limit the keyword to well-being/wellbeing, excluding the long list of related elements (e.g., stress, burnout, and satisfaction). One might argue that such a criterion downgrades the comprehensiveness of the findings. Nonetheless, using well-being/wellbeing only was not intended to exclude studies employing other elements. Rather, it was intended to include authors who explicitly focused on teacher well-being, even though they chose other dimensions/elements to represent well-being in the study. It should be emphasised that different starting points inevitably pave the way for a spread of papers with disparate conceptualisations, research purposes, questions, and designs, hence leaving a question mark for the eligibility of papers where well-being was not mentioned in the pool of well-being research.

## 8. Conclusions

ECE has been critical for children’s development throughout their lives. The well-being of ECE teachers has attracted more attention as it affects the development of teachers, young children, and even schools. It appears that the literature on ECE teacher well-being has accumulated a certain amount of research evidence. The publication rate has been accelerating; nevertheless, the field is still generally under-developed at the current stage. Notably, although publications from more developed regions continue to predominate in terms of both quantity and academic focus, some developing regions are making significant progress in narrowing the gap. This review also reveals the imbalances in research foci, research types, research methods, and data collection techniques. More precisely, antecedents of well-being, empirical studies, and quantitative methods along with certain techniques have consistently attracted the attention of researchers. In response to this situation, this review identifies several future directions. For instance, regarding research types, while conceptualisation of teacher well-being remains elusive, more conceptual discussions should be planned. Additionally, greater diversity in research designs and techniques is needed, not only to address existing gaps in quantity but also to capture more accurate and unique experiences of ECE teachers. The authors hope that this review inspires more stakeholders to pay more attention to the importance of teacher well-being, especially when facing vulnerable young children. It is also suggested that all stakeholders, including scholars, practitioners, and policymakers, modify their research and practices to not only contribute to a more mature knowledge base for the field but also embolden ECE teachers to thrive rather than simply survive and recover during unprecedented times.

## Figures and Tables

**Table 1:** Data Collection Locations in Five-Year Segments From 1993 to 2023.

Range	USA	Mainland China	HK	Australia	Finland	South Korea	Ghana	Italy	New Zealand	Spain
1993–1997	1	0	0	0	0	0	0	0	0	0
1998–2002	0	0	0	0	0	0	0	0	0	0
2003–2007	0	0	0	1	0	0	0	0	1	0
2008–2012	1	0	1	0	3	0	0	0	0	1
2013–2017	9	0	3	3	1	0	0	1	0	0
2018–2023	54	18	12	9	5	6	5	4	2	2
Total	65	18	16	13	9	6	5	5	3	3

Note. n = 158.

**Table 2:** Publication Numbers by Themes From 1993 to 2023.

Nature	No. and %	Antecedent	No. and %	Effect	No. and %
Theory	5, 3.1%	Contextual	24, 15.1%	Teachers’ personal outcome	24, 15.1%
Definition	3, 1.9%	School-level	94, 59.1%	Teaching practices	13, 8.2%
Content	19, 11.9%	Personal	78, 49.1%	Students’ learning processes	18, 11.3%
Measures	3, 1.9%	-	-	Students’ academic outcome	5, 3.1%
		-	-	School-level outcomes	3, 1.9%
Total	29, 18.2%	-	128, 80.5%	-	48, 30.2%

Note. n = 159.

**Table 3:** Qualitative data collection techniques in five-year segments from 1993 to 2023.

Range	Interviews	Classroom Observations	Focus Groups	Qualitative Surveys	Case Studies	Journal Writing	Life Experience	Reflective Reports
1993–1997	0	0	0	0	0	0	0	0
1998–2002	0	0	0	0	0	0	0	0
2003–2007	1	0	1	0	0	0	0	0
2008–2012	1	1	1	0	0	0	0	0
2013–2017	4	4	2	0	1	0	0	0
2018–2023	19	7	5	4	2	1	1	1
Total	25	12	9	4	3	1	1	1

Note. n = 53.
